# Complications and Risk Factors in En Bloc Resection of Spinal Tumors: A Retrospective Analysis on 298 Patients Treated in a Single Institution

**DOI:** 10.3390/curroncol29100620

**Published:** 2022-10-17

**Authors:** Stefano Bandiera, Luigi Emanuele Noli, Cristiana Griffoni, Giovanni Tosini, Elisa Carretta, Stefano Pasini, Eleonora Pesce, Alfio Damiano Ruinato, Giovanni Barbanti Brodano, Giuseppe Tedesco, Marco Girolami, Silvia Terzi, Riccardo Ghermandi, Gisberto Evangelisti, Valerio Pipola, Alessandro Gasbarrini

**Affiliations:** 1 Department of Spine Surgery, IRCCS Istituto Ortopedico Rizzoli, 40136 Bologna, Italy; 2 Department of Programming and Monitoring, IRCCS Istituto Ortopedico Rizzoli, 40136 Bologna, Italy

**Keywords:** spinal tumors, en bloc resection, complications, overall survival

## Abstract

En bloc resection consists in the surgical removal of a vertebral tumor in a single piece with a sufficient margin, to improve survival and reduce recurrence rate. This procedure is technically demanding and correlates with a high complication rate. The purpose of this study is to investigate the risk factors for complications in en bloc resection and evaluate if benefits overcome the risks in term of overall survival. We retrospectively analyzed prospectively collected data of patients treated with en bloc resection between 1980 and 2021. Complications were classified according to SAVES-V2. Overall Survival was estimated using Kaplan-Meier method. A total of 149 patients out of 298 (50%) suffered from at least one complication. Moreover, 220 adverse events were collected (67 intraoperative, 82 early post-operative, 71 late post-operative), 54% of these were classified as grade 3 (in a severity scale from 1 to 6). Ten years overall survival was 67% (95% CI 59–74). The occurrence of relapses was associated to an increased risk of mortality with OR 3.4 (95% CI 2.1–5.5), while complications did not affect the overall survival. Despite a high complication rate, en bloc resection allows for a better control of disease and should be performed in selected patients by specialized surgeons.

## 1. Introduction

Primary bone tumors of the spine are very rare, making up around 5% of all primary bone tumors [1].

Conversely, there are approximately 180,000 new cases of metastatic involvement of the vertebral column annually in the USA, and up to one-third of all patients with cancer develop metastases to the spinal column [2].

En bloc resection of primary tumors refers to the surgical removal of the whole tumor in a single piece, fully encased by a layer of healthy tissue. This procedure was first proposed by Enneking et al. [3] for bone and soft-tissue limb tumors, possibly in combination with adjuvant chemotherapy within the so called ‘‘limb salvage surgery.’’ The new procedure dramatically reduced the rate of amputations without the negative impact of this procedure and sometimes even improving the local and systemic disease control.

Stener [4] was the first pioneer of en bloc resections in the spine, followed later on by Roy-Camille et al. [5], and finally by Tomita et al. [6]. According to histologic diagnosis and oncological staging system, en bloc resection in the spine is indicated for aggressive benign tumors (Stage 3), malignant primary tumors and some isolated metastases such as renal cell carcinoma [7]. Adjuvant treatments should also be considered for high-grade malignancies.

The Weinstein-Boriani-Biagini (WBB) surgical staging system [8] was created for planning en bloc resection to achieve the appropriate histologic margin, significant for prognosis [9]. These operations can also be performed in the spine, where anatomical and surgical constraints make them technically demanding. Multiple approaches or a widely enlarged single posterior approach must be planned to increase the complexity of the surgery, and therefore the risk of complications. The ambitious goal to achieve oncological wide margins sometimes means the resection of anatomical structures (dura, pleura, nerve roots, muscles, and vessels), with the drawback of worse morbidity and functional results.

The main purpose of this procedure is a low recurrence rate and increase in survival.

The so called “radical resection” in the spine is limited due to the neighboring noble structures, which cannot always be sacrificed, and this negatively affects the local control of the disease.

Perioperative treatments such as radiation therapy and chemotherapy improve oncological results in some tumors, but they are also cause of local complications (wound dehiscence and infection) [10].

The aim of this study is to analyze complications in en bloc resection and their impact on survival, comparing it with the impact of recurrences, in order to understand how the benefits outweigh the risks when we candidate a patient to this surgery.

## 2. Materials and Methods

This is a retrospective analysis of data prospectively collected as part of an observational study approved by the Institutional Ethics Committee of Istituto Ortopedico Rizzoli on 14 December 2016 (protocol number 0022814), concerning the retrospective and prospective collection of clinical and radiographic data related to spinal diseases (of degenerative, oncological, traumatic and infectious origin).

The research was performed according to the Declaration of Helsinki. The signature of a study-specific informed consent was obtained from patients prospectively enrolled for the study, but it was not required for retrospective data due to the regulations taking place in health institutions dedicated to scientific research.

Patients with spine tumors treated with en bloc resection by the same team of surgeons between December 1980 and May 2021 were retrieved for analysis. The indication for en bloc resection was given considering the histology, the staging, the surgical classification and patient’s clinical condition and prognosis. All patients underwent a complete radiographic examination and staging assessment and were classified according to the Enneking [11] and Weinstein-Boriani-Biagini (WBB) staging systems [8].

Patient outcomes were retrospectively evaluated using parameters associated to the disease and parameters associated to the surgery. Parameters related to the disease included local recurrence and mortality, while parameters arising from the intervention included intraoperative and post-operative surgical complications (SC) and adverse events (AEs).

SCs and AEs were collected using McDonnell classification [12] and retrospectively classified with the Spinal Adverse Events Severity System (SAVES-V2) [13] following an accurate revision of electronic medical records.

McDonnell classification classifies severity in “major” and “minor”, with “major” evaluating an AE for which an increase in hospitalization days (LOS) is expected and “minor” for which it is not.

SAVES-V2 classifies SCs and AEs as intraoperative and post-operative; it evaluates their estimated effect on the LOS and grades their severity on a scale ranging from grade 1 to grade 6, where grade 1 indicates AEs that does not require any treatment, conversely grade 6 AEs resulting in death (Table 1). We decided to perform a further division in “early post-op” (AEs that occur in the first 30 days after surgery) and “late post-op” (after 30 days from surgery). A more specific temporal distribution of AEs’ incidence could help clinicians managing patients during hospitalization and allows to estimate the influence of surgical AEs on patients’ post-operative clinical outcomes and on the economic impact of the surgery.

Several risk factors were evaluated for their impact on the overall survival and on the complication rate, including age, gender, surgical approach adopted (single posterior or combined anterior and posterior), previous surgical treatment of the tumor and other treatments before index surgery (chemotherapy (CHT) or radiotherapy (RT), or both).

### Statistical Analysis

Data were summarized by using frequencies and percentages for categorical variables and by means of median and range for the continuous variables. Groups were compared using the Chi-square or Fisher Exact test, as appropriate. The Kaplan-Meier method was used to estimate overall survival (OS) functions. The log-rank test was used for group comparison. OS was defined as the time since surgery until death or last follow-up examination. Median follow-up time was computed using the reverse Kaplan-Meier estimator. The Cox proportional hazards model was used to compute hazard ratios and 95% confidence intervals (Cis) of potential prognostic factors for overall survival. While the logistic regression was used to compute odds ratios and 95% confidence intervals (Cis) of potential prognostic factors for the risk of relapse and the risk of complication. The correlation between variables was investigated to derive the final multivariate prognostic models. Moreover, the choice of variables to include in the final models was made, taking into account a 10% statistical significance in univariate analysis. Analyses were performed using SAS 9.4 (SAS Institute Inc., Cary, NC, USA).

## 3. Results

From December 1980 to May 2021, our team performed a total of 327 en bloc resections for spinal tumors. AEs and SCs evaluated with McDonnell classification showed that 99 patients had at least one major complication and 58 had at least one minor complication. Overall, 157 patients out of 327 (48%) suffered for at least one complication.

For twenty-nine patients, data retrieved from electronic medical records were insufficient to apply SAVES-V2 classification system, so these cases were removed from all the analysis and our study included 298 patients.

As reported in Table 2, the study group consisted of 163 male and 135 female patients with an average age of 48 years (range 3–82).

Most of tumors treated by en bloc resection were malignant primary tumors (59%), while 24% were spinal metastases and 16% were benign primary tumors (Table 3).

Main lesions were more frequently localized in the thoracic spine T3–T11 (104 lesions, 35%), in the lumbar spine L2–L4 (102 lesions, 34%) and in the thoraco-lumbar tract T12–L1 (57 lesions, 19%) (Table 2).

We observed that 83 patients (27.8%) received a previous treatment including radiotherapy and/or chemotherapy at the main lesion site before en bloc resection, and 47 patients (15.8%) received a previous surgical treatment at the main lesion.

### 3.1. SAVES-V2 Classification of Complications

Complications were observed in 149 out of 298 patients (50%) for a total of 220 complications.

As reported in Table 4, Table 5 and Table 6, 60 of 298 patients (20%) had intraoperative complications, 65 patients (22%) had early post-operative complications and 64 patients (21%) had late post-operative complications.

A total of 28 of 298 patients (9.4%) had both intraoperative and post-operative complications; 21 patients of 298 (7.5%) had early and late post-operative complications and 9 patients had all three types of complications (3%).

**Figure 1:**
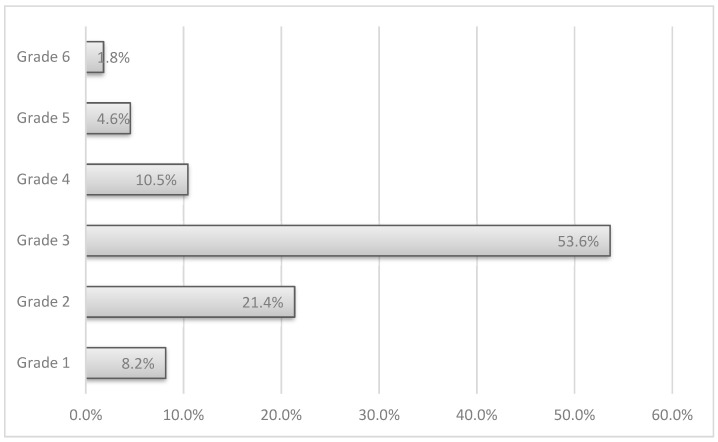
Adverse Events severity grade distribution. Graph shows the distribution of AEs by grades according to SAVES v2 system. “Grade 3” AEs are the most common in our cohort: 53.6% of events are likely to have a temporary effect on outcome.

The severity grade of complications was evaluated according to SAVES system reported in Table 1. In our study cohort (Figure 1), most of the complications (54%) had a severity grade 3, which indicates adverse events requiring an invasive (e.g., surgery) or complex treatment (e.g., monitored bed) and have a temporary (<6 months) adverse effect on outcome.

Overall, 4 out of 149 complications led the patients to death. In two cases, the death was due to intraoperative AEs (vascular and visceral injuries, respectively). In one case the patient died a few days after surgery as a result of an intraoperative bleeding. The other two deaths occurred in the early post-operative period and were both caused by heart failure as a consequence of pulmonary-related AEs.

Concerning intraoperative complications (Table 4), 54 patients had one complication and 6 patients had two complications, with a total of 67 complications. Furthermore, 9% of intraoperative complications had severity grade ≥4 (major complications) and two of them were fatal.

The most frequent intraoperative complications were: dural tear (23/67, 34%), visceral injury (12/67, 18%), vascular injury (10/67, 15%), hardware malposition requiring revision (6/67, 9%), anesthesia-related complications (4/67, 6%), nerve root injury (3/67, 4.5%).

Concerning early post-operative complications (Table 5), 46 patients had one complication, 13 patients had two complications and 6 patients had three complications with a total of 82 complications. Moreover, 24% of early post-operative complications had severity grade ≥4 (major complications) and two of them were fatal.

The most frequent early post-operative complications were hematoma (10/82, 12%), wound-related complications (6 deep wound infections + 4 would dehiscence, 10/82, 12%), CSF leak/meningocele (8/82, 10%), pulmonary embolism (7/82, 8.5%), neurologic deterioration ≥1 motor grade in ASIA motor scale (6/82, 7.3%). There was also a large number of complications classified as “others” (33%).

Concerning late post-operative complications (Table 6), 56 patients had one complication, 7 patients had two complications and 1 patient had three complications with a total of 71 complications; 15% of late post-operative complications had a severity grade ≥4 (major complications).

Mechanical failure was the most frequent late post-operative complication, associated with loss of correction in 18 cases (18/71, 25%) and without loss of correction in 22 cases (22/71, 31%); other frequent complications were wound-related complications (6 deep wound infection and 5 wound dehiscence, 11/71, 15.5%). There was also a large number of complications classified as “others” (17%).

Post-operative complications as a whole (early + late) represent 70% of all complications recorded in our cohort of patients treated by en bloc resection.

### 3.2. Analysis of Survival and Complications

**Figure 2:**
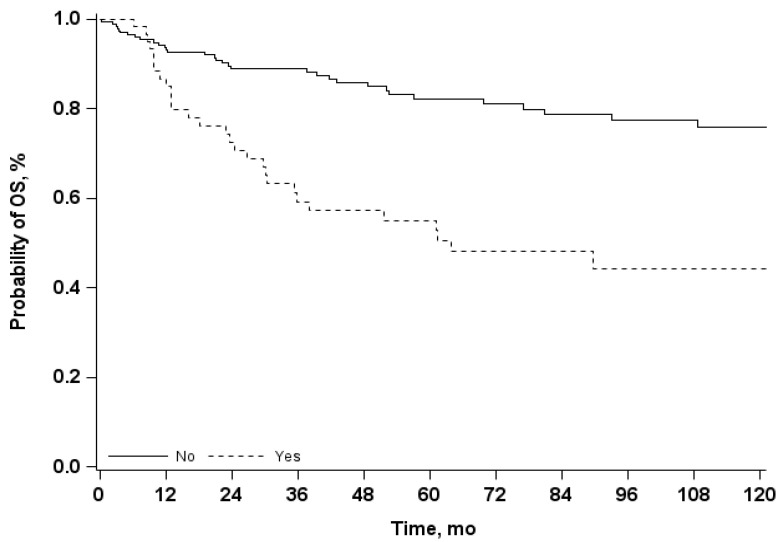
Overall Survival (OS) Kaplan-Meier curves in patients with and without relapse; mo = months.

**Figure 3:**
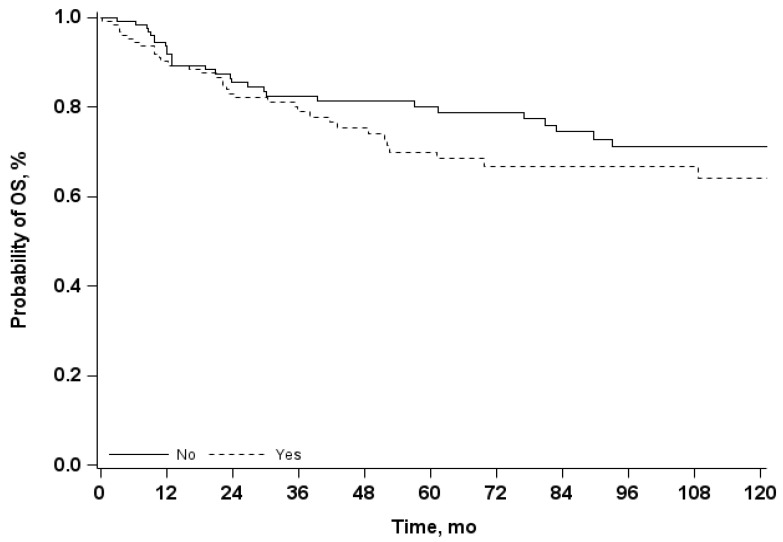
Overall Survival (OS) curves in patients with and without complications; mo = months.

**Figure 4:**
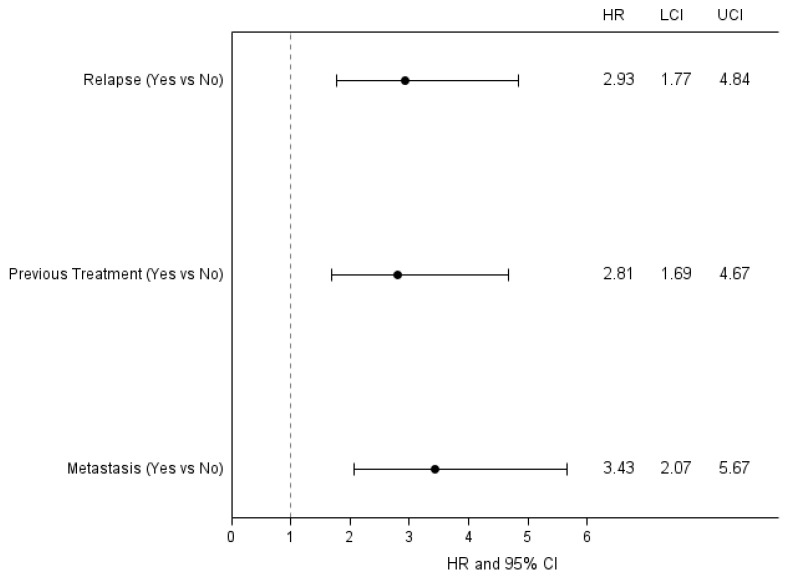
Forest plot for Overall Survival in association with prognostic factors significant in the multivariable model.

Median follow-up time was 69 months (IQR 28–127 months). The Kaplan-Meier estimates of 5-years OS was 75% (95% CI 69–81) and 10-year OS was 67% (95% CI 59–74). Using the univariate Cox model, we observed that the presence of a relapse is associated to an increase risk of mortality, with an OR 3.4 (95% CI 2.1–5.5) (Table 7 and Figure 2), while the presence of complications does not affect significantly the OS (Table 7 and Figure 3). Results from multivariate analysis confirmed that the presence of a relapse had a significant impact on survival, in addition to the presence of a previous treatment and of a metastatic tumor (Figure 4).

Regarding the risk factors for complications (Table 8), through a univariate analysis we observed that the occurrence of complications is significantly affected by previous treatments (OR 2.8, 95% CI 1.7–4.9), previous surgery (OR 2.5, 95% CI 1.3–5.0) and double surgical approach (longer and more invasive surgery) (OR 2.0, 95% CI 1.2–3.3).

**Figure 5:**
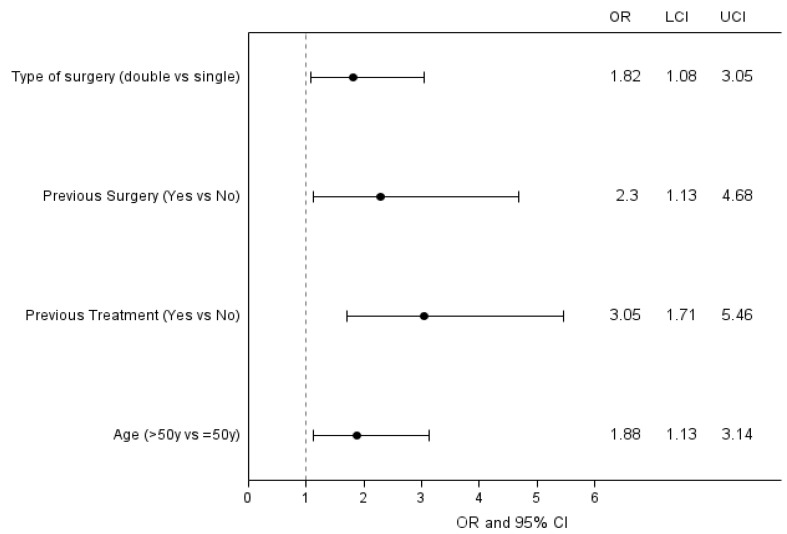
Forest plot for Complications in association with prognostic factors significant in the multivariable model; y = years.

The multivariate analysis reported in Figure 5 confirms the association of complications with the type of surgery, previous treatments and previous surgery, but also age higher than 50 years old resulted to be as a risk factor in this model.

This section may be divided by subheadings. It should provide a concise and precise description of the experimental results, their interpretation, as well as the experimental conclusions that can be drawn.

## 4. Discussion

En bloc resection represents the surgical procedure aiming to remove entirely primary aggressive neoplasms [6,14,15] or selected metastatic lesions [15,16] (such as solitary radioresistant tumor), alongside with a free-of-disease healthy tissue surrounding the mass, known as “margin”.

En bloc resection, by its own definition, aims to be oncologically adequate, since the tumor mass should not be violated and no disease should be left out of the resection.

As consequence, to reduce at the very minimum the risk of local recurrence and achieve local and systemic control over the disease, a wide margin must be obtained whenever possible.

Due to a more challenging surgery compared to conventional resection techniques, every surgeon has to take into account morbidity associated to en bloc resection [17,18]. It is important to stress that since specific skills and toolsets are required, there is a concrete risk that non-specialized centers could perform inadequate excisions, leading to the necessity of a second intervention to achieve an adequate resection.

Araujo et al. [19] reported in their 5-years of experience a complication rate of 76%; Zhang et al. [20] reported a case series of 31 patients over an 8-year period treated for thoracic tumors invading the spine, where at least one complication occurred in 50% of cases with a major complication rate of 32.3%. Our team studied morbidity of en bloc resection during the last two decades reporting data on complications occurring in a large series of patients affected by primary spine tumors surgically treated in our tertiary referral center [14,17,18,21,22,23]. A systematic review concerning perioperative complications in en bloc resection for spinal tumors was published ten days ago [24]. It included thirty-six studies with 961 patients, describing the more frequent complications occurring in en bloc resection.

The present study is a retrospective analysis of prospectively collected data and it represents an updated version of the study published by Boriani and co-workers in 2016 [21]. This paper analyzed a cohort of 220 patients surgically treated by en bloc resection from 1990 to 2015 and reported a complication rate of 45%. From 2015, 104 new patients underwent en bloc resection; before 1990, 3 patients underwent surgery: both were added to the current analysis, increasing the percentage of patients with complications to 48% in the entire cohort (1980–2021). Thus, our series of patients, treated at the same Institution during the last thirty years, accounts for one third of all patients included in 36 studies recently analyzed [24].

The improvement of surgical techniques, gained experience, new and targeted oncological therapies would have suggested a decrease in AEs over the years. To monitor the temporal trend, we assessed the number of patients with complications recorded over the years, using year 2015 as cut-off time: before 2015, 45% of patients (89 out of 199) had at least one complication, while this percentage increased to 65% (59 out of 99) for patients treated after 2015. Despite the fact that this complication rate is consistent with the literature [19,20,24], we analyzed in detail our cohort study to better understand the reasons underlying the complication incidence. In fact, in the last decades the high rates of complications recorded in spine surgery prompted to pay particular attention on this topic, considering the high impact on patients’ quality of life and the relevant costs for healthcare systems. First of all, new systems for the capture and classification of complications were implemented, encouraging clinicians to a more systematic and standardized collection of data concerning AEs.

Concerning our cohort, from 1990 to 2015, 23.8% of patients (54/227) had already been treated with CHT, RT or surgery; from 2015 to 2021 were 30% (30/100). In the same cohort, before 2015, 30.2% of patients had tumors rated “extra-compartimental” (IB, IIB, III) in the Enneking system; after 2015, this percentage increased to 50.5%. We can assume that some patients that previously were considered ineligible for en bloc resection because of the high risks associated to the procedure, in the last ten years were subjected to en bloc resection, probably due to the increased experience of spine surgeons in our team. Previous treatments and previous surgery have been recognized as risk factors in complication incidence; extra-compartimental tumors need more aggressive resection as well, with an increased risk of AEs occurrence. Taken together, a more accurate capture of complications and worst baseline characteristics of patients subjected to an aggressive surgery might explain why AEs did not decrease as expected. In order to compare the outcome in the two cohorts of patients treated before and after 2015, we performed a Kaplan-Meier analysis of 5-year OS and observed no significant difference.

For a more accurate analysis we retrospectively re-assessed the same cohort (1980–2021, excluding 29 patients) with SAVES-V2, which nowadays represents the most used and reliable classification system for identifying and capturing AEs in spinal surgery [13]. The vast majority of complications reported in our experience were classified as grade 3 according to SAVES-V2 severity scale, which means they required invasive or complex treatments but are most likely to have a temporary (<6 months) adverse effect on outcome. A total of 4 patients out of 298 (1.3%) died because of adverse events.

### 4.1. Intraoperative Complications

Concerning the intraoperative complications, dural tear was the most common AE, affecting 23 patients on 298 (7.7%). In spinal surgery, durotomy occurs with an incidence ranging from 0.2% up to 20% [25]. Several risk factors have been considered explaining a high rate of dural lesions, such as age, sex and revision surgery [25], with studies showing that dural tears were likely to occur more than twice in patients who have been surgically treated in the same area [26]. It should be taken in account that 15.8% (47/298) of our patients has already received surgery before en bloc resection and this number has increased constantly in the last few years. Scar tissue from prior surgery surrounding the dural sac can result in dural adhesion and obscure the anatomy; the same effect can be due to radiation-induced fibrosis. The aggressive nature of en bloc surgery prompts surgeons to treat the tumor with wide margins. For intra-canal tumors in particular, how many of dural tears could be effectively considered adverse events and how many were inevitable events occurring to completely remove the tumor should be investigated. Most of these dural tears were detected and treated within the surgery, with only two Cerebrospinal fluid leak as direct consequence of these evident dural lesions.

The second and third most common intraoperative adverse events were visceral and vascular injuries: 10 vascular injuries occurred (10/298 patients, 3.4%) and 5 of them were lesions of the inferior vena cava, in patients who received a double approach (anterior + posterior) for particularly invasive tumors that could have incorporated vessels and other non-spinal structures. Generally, the risk of vascular injuries and extensive bleeding was reduced by combined approach as it allows for the resection of anterior structures under direct visual control.

Concerning visceral injuries, most of them were pleural lesions in patients with tumor rated IB and IIB (extracompartimental in both cases) in the Enneking scale. This category of complication is associated to the localization and the extension of the tumor which can involve visceral structures.

We also observed 6 cases of hardware malpositioning which required revision (6%298 patients, 2%), which is lower rate in comparison to other reports [24].

Concerning neurological damages, we observed 3 cases of nerve root injuries (3/298 patients, 1%) and 6 cases of early post-operative neurological deterioration (6/298 patients, 2%). This rate is lower than that reported in the systematic review recently published by Li et al. [24]. The authors recommend the use of neuromonitoring during en bloc resection procedures, which is adopted at our Institution.

### 4.2. Early Post-Operative Complications

Regarding early post-operative complications, the most common AE reported in the first 30 days after the surgery was “hematoma” followed by “Cerebrospinal Fluid leak” and “pulmonary embolism”.

The most common cause of a post-operative CSF leak is a dural tear and a direct suture repair of the breakage should always be performed, but still it has a failure rate of 5% to 9% [27]. When unproperly treated or unnoticed, CSF leak may lead to serious sequelae. One CSF leak occurred in a patient with an intersomatic cage that damaged the dura; in the remaining CSF leak cases the original cause could be attributed to undetected dural tear [28], treated as soon as noticed.

A lot of post-operative adverse events were rated as “other”, since a precise classification within SAVES was not possible. Most of them were airway-related diseases (chylothorax, pneumothorax, pleural effusion, respiratory failure) and other systemic issues (allergic reaction, organ and multi-organ dysfunction/failure).

### 4.3. Late Post-Operative Complications

Regarding late post-operative adverse events, we registered 40 construct failures with or without loss of correction. These kind of events are quite common in spinal surgery due to the complexity of the kinematic of the spine. The risk of hardware failure is higher in patients undergoing en bloc resection than in patients undergoing palliative intralesional resection surgery. In oncological spine surgery, pre-operative and post-operative treatments, in particular radiotherapy, affect health tissues too with a negative impact on bone quality and recovery process, leading to problems in fusion and mechanical seal of tissues. En bloc resection in oncological patients requires a massive removal (tumor and healthy tissues as well), with consequences for the mechanical stability of the spine. The sagittal spino-pelvic alignment following en bloc resection has recently been analyzed [29].

Hardware failures include smaller issues, such as screws loosening, that usually require only monitoring, as well as more severe problems, from rods breakage to cage mobilization, that are much more likely to require a revision surgery. These failures with loss of correction are often paired with infections and wound dehiscence, the third and fourth most common late post-operative AEs in our records. Wound infection has been previously detected as the most common complication in patients undergoing spinal surgery for oncological diseases [30]. Deep wound infections are less likely to occur in minimally invasive spine surgery than in major surgery (1.5% vs. 3.8%) as prolonged operative time can increase the risk of intraoperative contamination [31]. Thus, a double surgical approach should be considered as a risk factor too: 8 out of 12 patients in our record that developed deep wound infection in the early or in the late post-operative period were surgically treated with double (anterior + posterior) approach, with an increased risk of contamination and wound complications. Moreover, the general status of oncological patients (inadequate nutritional condition and immunosuppression caused by the illness or by treatments) can be involved in wound-related complications.

### 4.4. Risk Factors

Several items have been considered as potential risk factors for complications’ onset.

An age higher than 50 was a predictive factor for AEs, even if the difference is not statistically significant (*p* < 0.055) for the univariate model. Elderly is usually related to degenerative disease of the spinal canal that might have a role favoring complications occurrence in spinal surgery [25] and the frailty of elderly population with oncological diseases should be taken into account.

Previous treatments (chemotherapy and radiotherapy) and previous surgery are associated to a statistically significant increase in risk of complications, explained by the impact of any of these treatments on tissues: fibrotic, traumatized and scar tissues together with a modified anatomy of vertebral structures could make healing harder. Moreover, previous surgery resulted to be associated with a higher risk of relapse (32.6% vs. 21.7%) even if not statistically significant (*p* = 0.11). It should be considered that patients who underwent multiple surgeries, systemic or radiation therapy are more likely to deal with more aggressive tumors, requiring more aggressive treatments. In this regard, the analysis suggests that double surgical approach (compared to the single one) is related to a doubled risk of complications. This observation has emerged in the literature since 1996 when McDonnell and co-workers analyzed perioperative complications of anterior procedures of the spine and highlighted that combined procedures (with anterior and posterior approach under the same anesthesia session) had a significantly higher rate of major complications than staged and isolated procedures [12]. More recent literature confirmed the association of combined procedures with a higher rate of major complications [17].

In conclusion, patients who did not receive any treatment before en bloc resection have half the risk of complications compared to who had already received treatments.

In this analysis, we also observed if the presence of intra-operative AEs could affect the onset of late complications. We found that patients suffering for intra-operative AEs are supposed to develop post-operative AEs with the same frequency of patients without intra-op AEs (41.9% of patients with intra-operative AEs had post-operative AEs too, and 35.6% of patients without intra-operative AEs had post-operative AEs). More interestingly, the mean number of post-operative complications per patient was 1.17 in the group of patients without intraoperative AEs and 1.92 in the group of patients with intraoperative AEs.

These data suggest that the occurrence of intraoperative AEs could facilitate the onset of post-operative complications in the same patients. Thus, clinicians should pay attention to the onset of post-operative complications and implement anticipatory strategies to prevent it and minimize consequences, in particular for patients suffering for complications during surgery who appear prone to developing more subsequent complications.

### 4.5. Overall Survival

It is important to understand risk factors to minimize the incidence and severity of complications (both for patients and healthcare systems), considering that, together with other predictable and unpredictable factors, en bloc resection itself can be considered as main cause of AEs. To achieve en bloc resection, surgical planning should take into account not only the functional sacrifices, but also the intrinsic morbidity of these procedures [21]. To better evaluate pros and cons of en bloc resection and the impact that this demanding procedure has on prognosis, we compared the overall survival (OS) in the groups of patients with or without complications and in the groups of patients with or without tumor relapse. The analysis showed that relapses affect the OS while complications had no effect on OS. This is a relevant point to be taken into account for surgical decision-making in the treatment of primary spinal tumors. Less invasive procedures (such as intralesional resection) are associated to a reduced risk of complications, at a cost of less local disease control and, as a consequence, worst prognosis. Thus, aggressive wide surgery should be performed when needed and feasible.

## 5. Conclusions

En bloc resection of primary spinal tumors or metastatic lesions is a challenging procedure which correlates with a high complication rate and, therefore, it should be carried out by specialized surgeons. Within a multidisciplinary management of spinal tumors, it is important to enhance integration between surgery and other treatments, focusing on timing and proper planning, to reduce the risks and maximize the efficacy of each treatment. Adjuvant therapies, such as chemotherapy for osteosarcoma and Ewing’s sarcoma or Denosumab for giant cell tumor [32], can facilitate en bloc resection, by reducing the tumor volume and inducing ossification, which makes the lesion margins more identifiable.

Further studies should investigate how complications and relapses impact on the quality of life of patients who underwent en bloc resection in order to generate more specific recommendations and guidelines and inform the patients correctly.

Despite all the risks, these findings confirm that en-bloc resection remains the gold standard surgical procedure for selected patients in order to achieve better survival rates and better local control of disease.

## Figures and Tables

**Table 1:** SAVES-V2 severity grades.

Severity of AE	Clinical Impact
1	AE does not require treatment & has no adverse effect
2	AE requires minor invasive (e.g., Foley catheter, nasogastric tube) or simple treatment but has no long-term effect
3	AE requires invasive (e.g., surgery) or complex treatment (e.g., monitored bed) & is most likely to have a temporary (<6 months) adverse effect on outcome
4	AE requires invasive (e.g., surgery) or complex treatment (e.g., monitored bed) & is most likely to have a prolonged (>6 months) adverse effect on outcome *
5	Significant neural injury (i.e., 1 or more grade deterioration in ASIA grade) or serious life- or limb-threatening event or any sentinel event ^†^
6	AE resulting in death

* Any AE with a functionally significant (i.e., patient reported) and most likely prolonged (>6 months) adverse effect on outcome, regardless of required treatment (e.g., nerve root injury that cannot be treated), should be Grade 4. ^†^ A sentinel event is an unexpected, serious life- or limb-threatening event(s) or any event (e.g., wrong level surgery) that necessitates a formal institutional review process and reporting as defined by your specific institution.

**Table 2:** Patients’ characteristics.

	All Sample (*N* = 298)
Age, median (range), years	48 (3–82)
Gender, No. (%)	
Male	163 (54.7)
Female	135 (45.3)
Histotype, No. (%)	
Maligno	177 (59.4)
Metastasi	72 (24.2)
Begnino	49 (16.4)
Enneking, No. (%)	
IA	19 (6.4)
IB	88 (29.5)
II	4 (1.3)
IIA	11 (3.7)
IIB	45 (15.1)
III	49 (16.4)
IIIA	3 (1.0)
IIIB	5 (1.7)
na	72 (24.2)
unknown	2 (0.7)
Main Lesion, No. (%)	
C1-C5	12 (4.0)
C6-T2	11 (3.7)
T3-T11	104 (34.9)
T12-L1	57 (19.1)
L2-L4	102 (34.2)
L5-S1	12 (4.0)
Previous Treatment, No. (%)	
No	215 (72.2)
Yes	83 (27.8)
Previous surgery, No. (%)	
No	251 (84.2)
Yes	47 (15.8)
Type of Surgery	
Double approach	185 (62.1)
Single approach	109 (36.6)
unknown	4 (1.3)

Note: na is not applicable, Enneking staging system is not detected in metastatic patients.

**Table 3:** Most frequent tumor histotypes in the study population.

Diagnosis	No. (%)
**Primary tumors**	
Chordoma	70 (23.5)
Giant Cell Tumor (GCT)	24 (8.1)
Chondrosarcoma (CHS)	23 (7.7)
Ewing Sarcoma	21 (7.1)
Osteosarcoma (OGS)	16 (5.4)
Osteoblastoma (OBS)	11 (3.7)
Peripheral chondrosarcoma	8 (2.7)
Central chondrosarcoma	7 (2.4)
Epithelioid Hemangioendothelioma	6 (2.0)
Fibrosarcoma	3 (1.0)
Mesenchymal Chondrosarcoma	2 (0.7)
Epithelioid Hemangioma	2 (0.7)
Hemangioendothelioma	2 (0.7)
Spindle cell sarcoma	2 (0.7)
Schwannoma	2 (0.7)
Malignant Schwannoma	2 (0.7)
Others	18 (5.4)
**Metastatic tumors**	
Hypernephroma mets	36 (12)
Breast cancer mets	6 (2.0)
Osteosarcoma mets	5 (1.7)
Lung cancer mets	4 (1.3)
Ewing sarcoma mets	3 (1.0)
Tyroid cancer mets	3 (1.0)
Uterus cancer mets	2 (0.7)
Colon cancer mets	2 (0.7)
Liposarcoma mets	2 (0.7)
Synovial sarcoma mets	2 (0.7)
Others	11 (3.3)

**Table 4:** Intra-operative complications.

Intraop AE Category	No. (%)
1. Allergic reaction	0 (0.0)
2. Anesthesia related	4 (6.0)
3. Bone implant interface failure requiring revision	0 (0.0)
4. Cardiac	1 (1.5)
5. Cord injury	0 (0.0)
6. Dural tear	23 (34.3)
7. Hardware malposition requiring revision	6 (9.0)
8. Hypotension (systemic <85 mm Hg for 15 min)	0 (0.0)
9. Massive blood loss (>5 L in 24 h or >2 L in 3 h)	3 (4.5)
10. Nerve root injury	3 (4.5)
11. Pressure sores	0 (0.0)
12. Vascular injury	10 (14.9)
13. Airway/ventilation	3 (4.5)
14. Visceral injury	12 (17.9)
15. Other (specify:)	2 (3.0)

**Table 5:** Early post-operative complications.

Early Postop AE Category	No. (%)
1. Cardiac arrest/failure/arrythmia	1 (1.2)
2. Construct failure with loss of correction	1 (1.2)
3. Construct failure without loss of correction	1 (1.2)
4. CSF leak/meningocele	8 (9.8)
5. Deep vein thrombosis	5 (6.1)
6. Deep wound infection	6 (7.3)
7. Delirium	0 (0.0)
8. Dysphagia	0 (0.0)
9. Dysphonia	0 (0.0)
10. Gastrointestinal bleeding	0 (0.0)
11. Hematoma	10 (12.2)
12. Myocardial infarction	2 (2.4)
13. Neurologic deterioration ≥ 1 motor grade in ASIA motor scale	6 (7.3)
14. Nonunion	0 (0.0)
15. Pneumonia	1 (1.2)
16. Postop neuropathic pain	0 (0.0)
17. Pressure sores	0 (0.0)
18. Pulmonary embolism	7 (8.5)
19. Superficial wound infection	1 (1.2)
20. Systemic infection	1 (1.2)
21. Urinary tract infection	1 (1.2)
22. Wound dehiscence	4 (4.9)
23. Other	27 (32.9)

**Table 6:** Late post-operative complications.

Late Postop AE Category	No. (%)
1. Cardiac arrest/failure/arrythmia	0 (0.0)
2. Construct failure with loss of correction	18 (25.4)
3. Construct failure without loss of correction	22 (31.0)
4. CSF leak/meningocele	2 (2.8)
5. Deep vein thrombosis	3 (4.2)
6. Deep wound infection	6 (8.5)
7. Delirium	0 (0.0)
8. Dysphagia	0 (0.0)
9. Dysphonia	0 (0.0)
10. Gastrointestinal bleeding	0 (0.0)
11. Hematoma	0 (0.0)
12. Myocardial infarction	0 (0.0)
13. Neurologic deterioration ≥ 1 motor grade in ASIA motor scale	0 (0.0)
14. Nonunion	0 (0.0)
15. Pneumonia	1 (1.4)
16. Postop neuropathic pain	0 (0.0)
17. Pressure sores	0 (0.0)
18. Pulmonary embolism	1 (1.4)
19. Superficial wound infection	1 (1.4)
20. Systemic infection	0 (0.0)
21. Urinary tract infection	0 (0.0)
22. Wound dehiscence	5 (7.0)
23. Other	12 (16.9)

**Table 7:** Results from univariate Cox model for OS.

		OR (95% CI)	*p* Value
**Age**	>50 vs. ≤50	1.1 (0.6–1.7)	0.8261
**Gender**	M vs. F	1.5 (0.9–2.4)	0.1243
**Metastasis**	Yes vs. No	3.5 (2.2–5.8)	<0.0001
**Previous treatment**	Yes vs. No	3.3 (2.0–5.3)	<0.0001
**Previous surgery**	Yes vs. No	1.3 (0.7–2.5)	0.4089
**Type of surgery**	double vs. single approach	1.5 (0.9–2.5)	0.1725
**Relapse**	Yes vs. No	3.4 (2.1–5.5)	<0.0001
**Complication**	Yes vs. No	1.3 (0.8–2.2)	0.2464

**Table 8:** Results from univariate Logistic models for Complication.

		OR (95% CI)	*p* Value
**Age**	>50 vs. ≤50	1.6 (1.0–2.5)	0.0551
**Gender**	M vs. F	1.0 (0.6–1.6)	0.9297
**Metastasis**	Yes vs. No	0.7 (0.4–1.2)	0.2347
**Previous treatment**	Yes vs. No	2.8 (1.7–4.9)	0.0002
**Previous Surgery**	Yes vs. No	2.5 (1.3–5.0)	0.0074
**Type of surgery**	double vs. single	2.0 (1.2–3.3)	0.0054

## Data Availability

Data supporting results can be obtained from the corresponding author.
